# A comparative study of nonlinear cleanup rules in resonator networks

**DOI:** 10.3389/frai.2026.1793314

**Published:** 2026-06-25

**Authors:** Calvin Yeung, Prathyush Poduval, Mohsen Imani

**Affiliations:** Department of Computer Science, University of California, Irvine, Irvine, CA, United States

**Keywords:** associative memory, bound-vector factorization, compositional representations, hopfield networks, hyperdimensional computing, nonlinear cleanup rules, resonator networks, vector symbolic architectures

## Abstract

Hopfield networks retrieve stored patterns through recurrent nonlinear updates, and later associative-memory variants broaden this design space through alternative nonlinear retrieval rules. Resonator networks use an analogous iterative procedure to solve vector-symbolic factorization: each factor estimate is updated by unbinding the current estimates of the other factors and applying a cleanup rule against the corresponding codebook. This perspective suggests a family of resonator networks indexed by the cleanup nonlinearity. We study four variants—sign cleanup, ReLU cleanup, polynomial cleanup, and softmax cleanup—and compare their factorization capacity, terminal failure modes, convergence behavior, empirical complexity, hyperparameter sensitivity, matched-dimensionality FHRR behavior, sign-projection choices, internal update noise, and neural-output decomposition on visual scene data. Across these evaluations, the cleanup nonlinearity changes both the capacity transition and the dominant failure mode. These results motivate treating resonator networks as a family of modern Hopfield-inspired factorization dynamics rather than as a single update rule.

## 1 Introduction

Hopfield networks are a foundational model of associative memory: a query is repeatedly updated until it approaches one of the stored patterns (Hopfield, 1982). In the classical bipolar model, this retrieval rule uses a sign nonlinearity, and the standard random-pattern storage capacity scales linearly with dimension. Later associative-memory models show that the retrieval nonlinearity is a meaningful design choice. Modern continuous Hopfield networks replace hard sign updates with softmax-weighted retrieval (Ramsauer et al., 2021); dense associative memories study higher-order nonlinear responses (Krotov and Hopfield, 2016); and universal Hopfield networks provide a broader formulation in which the nonlinear stage of associative retrieval can be varied (Millidge et al., 2022).

Vector Symbolic Architectures (VSAs), also known as Hyperdimensional Computing (HDC), use high-dimensional vectors and algebraic operations such as binding and bundling to represent compositional structure (Kanerva, 2009; Frady et al., 2020). A central computational problem in these systems is factorization: recovering the constituent symbols of a bound vector from a search space that grows exponentially with the number of factors. Resonator networks address this problem through an iterative search-in-superposition procedure in which each factor estimate is repeatedly unbound from the current estimates of the remaining factors and then cleaned up against its codebook (Frady et al., 2020; Kent et al., 2020).

A traditional resonator uses a sign-based cleanup rule analogous to the classical Hopfield nonlinearity, but the analogy also suggests resonator variants associated with other nonlinear associative-memory updates. In particular, softmax, ReLU, and polynomial responses can be substituted for the original cleanup rule, yielding a family of modern Hopfield-inspired resonator dynamics. When the number of factors is one, resonator factorization reduces to single-codebook associative retrieval; for multiple factors, the updates become coupled through the unbinding operation.

Recent resonator work already considers complex-valued representations, neuromorphic implementations, and alternative nonlinearities in the cleanup stage (Renner et al., 2024). (Langenegger et al. 2023) further analyze in-memory factorization and show that device-level stochasticity can aid factorization in some hardware regimes. Our goal is complementary: we organize sign, ReLU, polynomial, and softmax resonator updates within a common cleanup-rule view and compare their behavior under a common experimental setting.

Our contributions are as follows:

- We formulate resonator factorization as a coupled family of modern Hopfield-inspired cleanup dynamics and make explicit that the one-factor case reduces to single-codebook associative retrieval.
- We compare sign, ReLU, polynomial, and softmax cleanup rules across factorization capacity, terminal failure modes, convergence, empirical complexity, hyperparameter sensitivity, and sign-projection choices.
- We extend this comparison to matched-dimensionality FHRR factorization and internal update noise, while separating correct convergence, spurious convergence, and non-convergence throughout the analysis.
- We evaluate the effect of cleanup rules on neural output decomposition on visual scenes.

## 2 Background

We first review the vector-symbolic factorization problem, then summarize the associative-memory perspective that motivates the nonlinear resonator variants studied in this work.

### 2.1 Vector symbolic architectures and factorization

At the core of HDC are vectors that represent discrete units (Kanerva, 2009). Each class of elementary symbols (which can denote different values of an attribute, for example) consists of a list of high-dimensional, holographic, and random hypervectors that preserve a predefined similarity function between different symbols within the class. The two common ways of sampling hypervectors are the bipolar representation and Fourier Holographic Reduced Representation (FHRR). Let *D* denote the vector dimension of a codebook. The similarity in both encodings is inherited from the complex Euclidean norm, defined as δ(*H, G*) = *H*^†^*G*/*D*, where the † denotes the complex-conjugate transpose.

In the bipolar representation, hypervectors are randomly sampled from {−1, 1}^*D*^, and the hypervectors representing different values of the same attribute are considered to be mutually orthogonal to each other. The FHRR representation, on the other hand, operates over a *d*-dimensional continuous feature space with feature vector *f*, to construct high-dimensional encodings that preserve a kernel similarity *K*(*f*−*G*) over the underlying feature space. This is done using the kernel trick (Rahimi and Recht, 2007), where a random *D*×*d* matrix *W* is sampled from a probability distribution *p*(ω), and the encoding is performed as *H*_*f*_ = exp(*iWf*). If *p*(ω) is chosen as the Fourier transform of a translation-invariant kernel *K*(*f*−*G*), then δ(*H*_*f*_, *H*_*g*_)≈*K*(*f*−*G*).

Thus, when attributes can take continuous values such as size, position, or shading, FHRR can be used for encoding data. This connection between randomized Fourier features, continuous variables, and VSA-style computation is also developed in the Vector Function Architecture literature (Frady et al., 2022). The set of all possible vectors for a given attribute is called the *codebook*. Given hypervectors $x_{1},\ldots ,x_{n}\in {ℍ}^{D}$ representing values of one attribute, the corresponding codebook is a matrix **X**∈ℍ^*n*×*D*^ whose *j*-th row is *x*_*j*_.

The three main operations in HDC, bundling, binding, and permutation, can be characterized by how they affect the similarity of hypervectors.

1. Bundling (+): Usually defined as element-wise addition. If *H* = *H*_1_+*H*_2_, then both *H*_1_ and *H*_2_ are similar to *H*.
2. Binding (*): Usually defined as element-wise multiplication. If *H* = *H*_1_*H_2_, then *H* is dissimilar to both *H*_1_ and *H*_2_. Binding also preserves similarity in the sense that δ(*H*_3_*H_1_, *H*_3_*H_2_)≃δ(*H*_1_, *H*_2_) (Frady et al., 2018).
3. Permutation (ρ): Usually defined as a rotation of vector elements. Generally, δ(ρ(*H*), *H*)≃0, so permutation can encode order in sequences.

**Figure 1:**
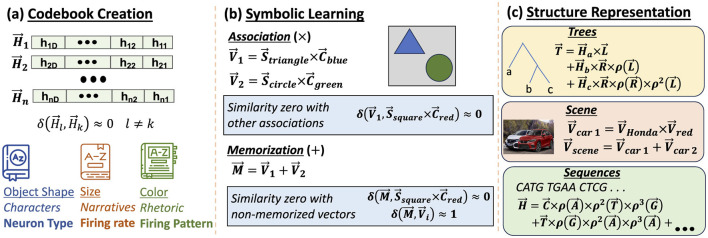
Overview of the operations in HDC and the representation of various data types. **(a)** Codebooks of hypervectors are initialized. **(b)** HDC respects respect symbolic operations of association and memorization. **(c)** HDC operations can be used to represent complex data structures.

Figure 1 summarizes these operations and their use in structured representations. A common construction is the “bind-and-bundle” scheme (Poduval et al., 2023, 2024), where the attributes of each object are first bound together and the resulting object vectors are bundled into a single memory vector. For example,


$$\begin{array}{l} \begin{array}{cc} h_{\text{scene}} & =h_{\text{hexagon}}*h_{\text{large}}*\cdots *h_{\text{light}}\\ +h_{\text{pentagon}}*h_{\text{small}}*\cdots *h_{\text{dark}}. \end{array} \end{array}$$ (1)

**Figure 2:**
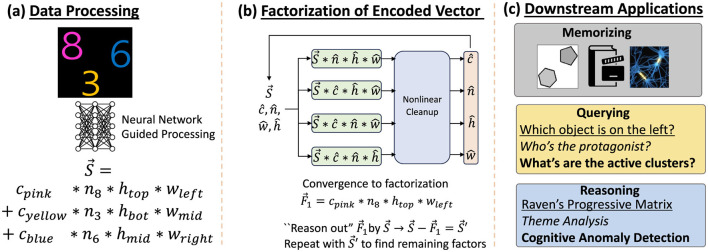
Schematic of resonator-based factorization for structured scene representations. **(a)** Scenes are mapped to structured HDC representations via a neural network; **(b)** This representation is factorized via resonator network; **(c)** The factors are used for downstream applications. These downstream examples are illustrative; the experiments in this paper evaluate backend factorization, not reasoning or explainability.

Factorization reverses the bound-vector construction: given a bound vector, the goal is to recover the corresponding codebook entries. For *F* factors and *n* values per factor, brute-force search scales as *M* = *n*^*F*^, motivating approximate iterative algorithms. Figure 2 visualizes a schematic of resonator-based factorization, the most common way of performing factorization on structured representations as in Equation 1.

### 2.2 Hopfield networks and nonlinear associative memory

A Hopfield network is an auto-associative memory that retrieves stored patterns by iterating an update map. In the classical bipolar setting, stored patterns ${\left\{\xi_{j}\right\}}_{j=1}^{n}$ with $\xi_{j}\in {\left\{-1,1\right\}}^{d}$ become attractors of the dynamics (Hopfield, 1982). Given a query $\xi_{t}^{0}$ and memory matrix **X** = [ξ_1_, …, ξ_*n*_], the standard update is


$$\begin{array}{l} \xi_{t+1}^{0}=\text{sgn}\left(\text{X}{\text{X}}^{⊤}\xi_{t}^{0}\right). \end{array}$$ (2)

The sign nonlinearity maps the superposed memory signal back into the bipolar state space in Equation 2. Later associative-memory variants modify this nonlinear stage. Modern continuous Hopfield networks use a softmax-weighted memory lookup,


$$\begin{array}{l} \xi_{t+1}^{0}=\text{X}\text{softmax}\left(\beta {\text{X}}^{⊤}\xi_{t}^{0}\right), \end{array}$$ (3)

where β is an inverse-temperature parameter in Equation 3 (Ramsauer et al., 2021). Dense associative memories study stronger nonlinear responses, including higher-order polynomial forms (Krotov and Hopfield, 2016), while universal Hopfield networks emphasize that associative-memory retrieval can be organized around a family of admissible nonlinear response choices (Millidge et al., 2022). We use this literature to motivate resonator variants that differ in their cleanup nonlinearity.

## 3 Methods

Let ${\text{X}}_{j}\in {ℍ}^{n\times D}$ denote the codebook matrix for factor *j*, with codewords stored as rows. Each factor estimate is initialized as a projected codebook superposition, ${\hat{x}}_{0}^{\left(j\right)}=\Pi_{j}\left({\text{X}}_{j}^{⊤}\text{1}\right)$, *j* = 1, …, *F*, where **1**∈ℝ^*n*^ is the all-ones vector.

At iteration *t*, define $r_{t}^{\left(j\right)}=s*\prod_{i\neq j}{\left({\hat{x}}_{t}^{\left(i\right)}\right)}^{-1}$, *j* = 1, …, *F*.

The generalized cleanup update is


x^t+1(j)=Πj,ϕ(Xj⊤ϕ(1DRe(X¯jrt(j)))),  j=1,…,F, (4)

where ${\bar{\text{X}}}_{j}$ denotes element-wise conjugation. We write Π_*j*, ϕ_ in Equation 4 to allow the post-reconstruction sign-projection choice to depend on the cleanup rule. In the bipolar experiments, the original sign rule and the softmax rule use coordinate-wise sign projection, while ReLU and polynomial cleanup omit sign projection and keep the reconstructed factor estimate as a real-valued superposition. The resulting state is decoded by nearest-codeword similarity, and unbinding uses the same element-wise multiplication used for bipolar states. Section 4.9 reports this ablation. For unit-modulus FHRR states, we retain the standard phase projection Π_*j*_(*z*) = *z*/|*z*| unless otherwise stated.

The cleanup rule is specified by the activation ϕ. We use ϕ_sign_(*a*) = *a*, ϕ_softmax_(*a*) = softmax(β*a*), $\phi_{\text{ReLU}}\left(a\right)=\mathrm{ReLU}\left(a\right)/\left[{\text{1}}^{⊤}\mathrm{ReLU}\left(a\right)\right]$, and $\phi_{\text{poly}}\left(a\right)=\mathrm{ReLU}{\left(a\right)}^{p}/\left[{\text{1}}^{⊤}\mathrm{ReLU}{\left(a\right)}^{p}\right]$, where powers are applied coordinate-wise. If the denominator is zero for ReLU or polynomial cleanup, the activation is set to uniform weights. With bipolar sign projection, ϕ_sign_ recovers the original resonator update up to the positive normalization by *D*. The ReLU and polynomial variants differ from the original sign resonator both in the rectified cleanup weights and, in the main reported setting, in the absence of bipolar sign projection.

## 4 Results

### 4.1 Setup

The experiments compare sign, softmax, ReLU, and polynomial cleanup rules. Unless explicitly labeled as an ablation, each rule is evaluated with its selected sign-projection choice: sign and softmax use bipolar sign projection, while ReLU and polynomial cleanup omit sign projection. Unless otherwise stated, each plotted condition uses 2,000 randomly sampled codebooks, maximum number of iterations *T*_max_ = 250, and early stopping when the factor estimates converge. Error bars show standard deviations over the randomly sampled codebooks. For the internal-noise experiments, error bars are computed across independently resampled codebook batches rather than from a single fixed batch.

For each trial, we sample codebooks **X**_1_, …, **X**_*F*_ of size *n*, select indices (*i*_1_, …, *i*_*F*_), and form $\text{s}={\text{x}}_{1}^{\left(i_{1}\right)}*\cdots *{\text{x}}_{F}^{\left(i_{F}\right)}$, with search space *M* = *n*^*F*^. After inference, each factor is decoded by nearest-codeword similarity. Accuracy is the fraction of trials in which all decoded indices match the generating indices.

A trial is labeled correct convergence when it converges and all factors are decoded correctly, spurious convergence when it converges to an incorrect factorization, and non-convergence when it does not converge before *T*_max_.

Softmax inverse temperature β and polynomial degree *p* are chosen on a validation grid at *D* = 1, 000, using validation search spaces near *M*∈{10^3^, 5 × 10^3^, 2 × 10^4^}. This sweep selects β = 20 for softmax and *p* = 2 for polynomial cleanup in the main experiments. The same validation procedure is used to choose whether sign projection is applied after cleanup. For bipolar decoding, each returned factor state is matched to the codeword with largest absolute similarity and flipped when its signed similarity is negative.

### 4.2 Accuracy across factor counts

**Figure 3:**
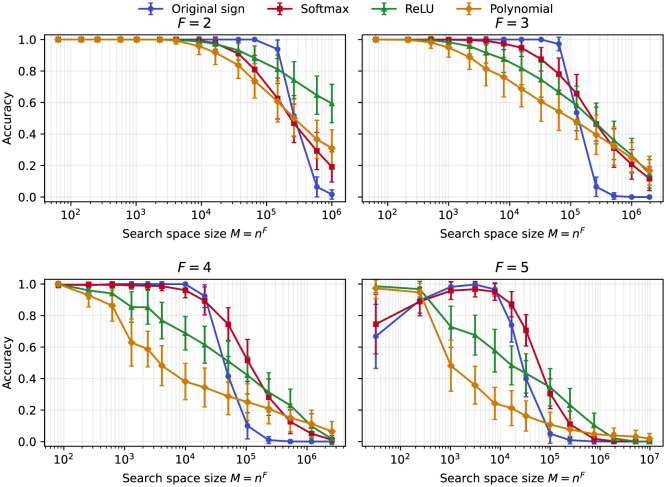
Accuracy across factor counts for bipolar codebooks with *D* = 1, 000.

Figure 3 reports accuracy as a function of search-space size for *F*∈{2, 3, 4, 5} using the selected sign-projection choice for each cleanup rule. All rules perform well at small search spaces, but their degradation patterns differ once the coupled factorization problem becomes harder. For *F* = 2, ReLU without sign projection has the most gradual high-*M* decline, while sign, softmax, and polynomial cleanup fall more sharply near the largest search spaces. For *F* = 3, sign and softmax remain near-perfect through moderate search spaces before dropping at large *M*, whereas ReLU and polynomial cleanup decline more gradually from lower search spaces. For *F* = 4 and *F* = 5, sign and softmax are strongest in the easier-to-moderate regimes, while the rectified rules without sign projection show smoother but not uniformly better degradation.

**Figure 4:**
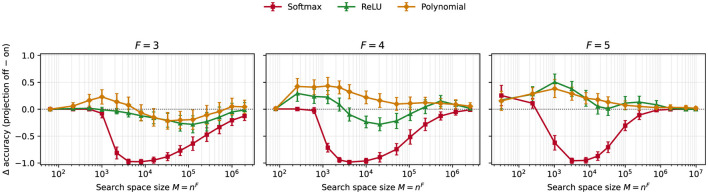
Effect of bipolar sign projection for softmax, ReLU, and polynomial cleanup with *D* = 1, 000 and *F*∈{3, 4, 5}. Each panel shows one factor count and plots the accuracy difference between omitting and applying sign projection, Δ = accuracy_off_−accuracy_on_. Positive values indicate that omitting sign projection improves accuracy. Marker shapes distinguish the cleanup rule.

These curves should therefore be read as a comparison between validated versions of the cleanup families, rather than as a comparison in which every rule is forced to use the same sign projection. The rectified rules already impose nonnegativity and normalization at the codebook-weight level; omitting sign projection preserves superposed reconstructed states that can remain useful for subsequent coupled updates. The benefit is not universal across all *F* and *M*, but the ablation in Figure 4 shows that sign projection is an important design choice for all cleanup rules. Overall, cleanup nonlinearities and sign-projection choices jointly determine how the coupled factorization dynamics degrade as *M* = *n*^*F*^ increases.

### 4.3 Terminal failure modes

**Figure 5:**
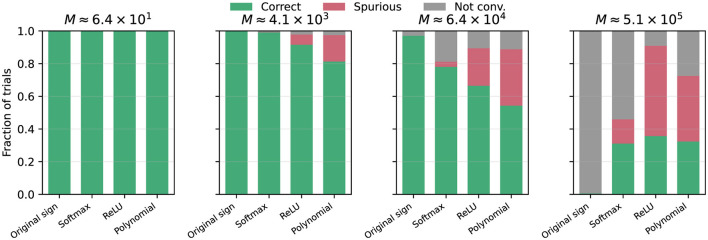
Terminal outcomes for bipolar codebooks with *F* = 3 and *D* = 1, 000.

Figure 5 decomposes the *F* = 3 bipolar experiments into correct convergence, spurious convergence, and non-convergence. This separation is important because the same accuracy value can correspond to qualitatively different dynamics. At small *M*, all cleanup rules primarily converge to the correct factorization. At intermediate search spaces, sign remains mostly correct, while the nonlinear cleanup rules begin to show different mixtures of spurious convergence and non-convergence. At the largest plotted search space, the sign rule primarily fails by not converging within the fixed iteration budget, whereas softmax, ReLU, and polynomial cleanup show a mixture of correct convergence, spurious convergence, and non-convergence.

The terminal classes therefore clarify the accuracy curves in Figure 3. In this setting, a drop in accuracy is not always caused by convergence to an incorrect attractor: it can also reflect dynamics that have not settled within *T*_max_ = 250. ReLU and polynomial cleanup without sign projection reduce some of the abrupt behavior seen when rectified reconstructions are binarized, but they can still converge spuriously when an incorrect partial factorization is amplified.

**Figure 6:**
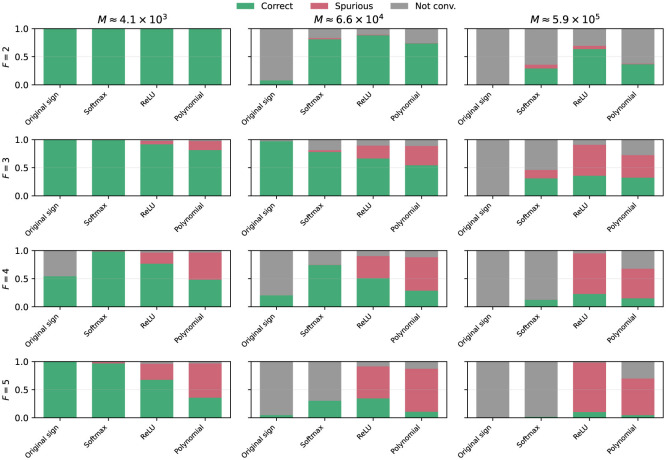
Terminal outcomes across factor counts for bipolar codebooks with *D* = 1, 000.

Figure 6 extends the terminal-outcome analysis across *F*∈{2, 3, 4, 5} and representative search-space sizes. The dominant failure mode changes with both factor count and cleanup rule. For smaller factor counts, failures are concentrated at larger search spaces and often appear as non-convergence for the sign rule. As the number of factors increases, failures appear earlier and become more heterogeneous: softmax tends to retain a large correct-convergence fraction in easier regimes, while ReLU and polynomial cleanup without sign projection often trade abrupt non-convergence for a mixture of correct and spurious convergence. This supports the view that factorization difficulty is not captured by accuracy alone. A complete evaluation should distinguish whether a method is confidently wrong, still searching, or correctly converged.

### 4.4 Neural-output decomposition on visual scenes

To assess whether cleanup-rule differences persist beyond exact synthetic bound vectors, we evaluate a learned-encoder decomposition setting on visual scenes. The experiment follows the interface used in neuro-vector-symbolic reasoning systems: a visual encoder maps an image to a VSA representation, and a resonator network module decomposes that representation into discrete factors for downstream reasoning (Hersche et al., 2023). We use Multi-CMNIST and Multi-dSprites, two multi-object abstract visual scene datasets. In Multi-CMNIST, each scene contains one to three colored MNIST digits (LeCun et al., 1998) on a 3 × 3 grid, with digit, color, horizontal-position, and vertical-position factors. In Multi-dSprites, each scene contains one to three dSprites objects, with shape, scale, orientation, horizontal-position, and vertical-position factors. For both benchmarks, the target scene representation is a bind-and-bundle vector, $s=\underset{i}{\sum }\prod_{f}x_{i,f}$, using normalized bipolar codebooks. A ResNet visual backbone initialized from ImageNet-pretrained weights is trained with a *D*-dimensional regression head to predict this scene vector using mean-squared error. The encoders produce high-quality VSA outputs, with test MSE/cosine of 0.0310/0.993 on Multi-CMNIST and 0.0643/0.988 on Multi-dSprites.

Our evaluation focuses on the decomposition stage of the neuro-vector-symbolic pipeline. Table 1 reports neural-output decomposition for each cleanup rule. Attribute accuracy measures individual factor recovery after object matching, object accuracy requires all factors of a matched object to be correct, and scene accuracy requires the full unordered object set to be recovered. Correct convergence is counted at the object-extraction level when the recovered bound object matches one of the remaining ground-truth objects; spurious convergence indicates convergence to a valid but incorrect object; and non-convergence indicates that the dynamics did not stabilize within *T*_max_ iterations.

**Table 1:** Neural-output decomposition on visual scenes.

Dataset	Cleanup rule	Attr. acc. ↑	Object acc. ↑	Scene acc. ↑	Correct conv. ↑	Spur. conv. ↓	Non-conv. ↓
Multi-CMNIST	Sign	0.981	0.971	0.952	0.570	0.010	0.421
Multi-CMNIST	Softmax	0.804	0.731	0.606	0.682	0.247	0.071
Multi-CMNIST	ReLU	0.911	0.890	0.803	0.889	0.095	0.016
Multi-CMNIST	Polynomial	0.795	0.745	0.581	0.747	0.233	0.020
Multi-dSprites	Sign	0.977	0.967	0.937	0.960	0.032	0.009
Multi-dSprites	Softmax	0.609	0.454	0.342	0.388	0.554	0.058
Multi-dSprites	ReLU	0.672	0.589	0.365	0.610	0.350	0.040
Multi-dSprites	Polynomial	0.503	0.345	0.141	0.371	0.601	0.029

A pretrained ResNet encoder is trained to output a bind-and-bundle VSA scene vector on Multi-CMNIST and Multi-dSprites; each cleanup rule then decomposes the continuous neural output.

The benchmark results show that cleanup-rule differences persist on learned visual outputs. Sign cleanup gives the strongest symbolic recovery on both datasets, reaching 95.2% exact scene accuracy on Multi-CMNIST and 93.7% on Multi-dSprites. ReLU cleanup is the next strongest rule on Multi-CMNIST and produces the highest correct-convergence fraction on that benchmark, but it degrades more sharply on Multi-dSprites. Softmax and polynomial cleanup have lower symbolic recovery, especially on Multi-dSprites, where their failures are dominated by spurious convergence.

### 4.5 FHRR evaluation

**Figure 7:**
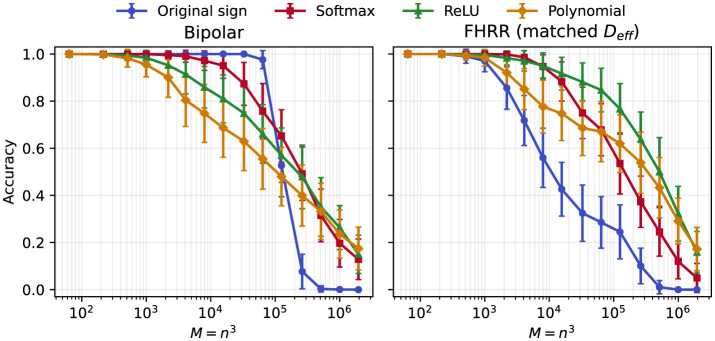
Matched effective-dimensionality comparison for bipolar and FHRR codebooks with *F* = 3 and *D*_eff_ = 1, 000.

Figure 7 compares bipolar and FHRR codebooks at matched effective real dimension. Because a complex FHRR coordinate has real and imaginary parts, the bipolar experiments use *D*_eff_ = 1, 000 and the FHRR experiments use *D*_eff_/2 = 500 complex entries. The bipolar panel follows the selected sign-projection choices described in Section 4.1, while the FHRR panel uses the corresponding phase projection for complex unit-modulus states.

The original complex cleanup is functional at small search spaces but degrades earlier than the nonlinear alternatives as *M* grows. ReLU has the most gradual FHRR degradation over much of the plotted range, while softmax and polynomial cleanup occupy intermediate regimes. The comparison also shows that the same cleanup rule need not behave identically across bipolar and FHRR representations, so representation type should be evaluated explicitly rather than treated as a direct implementation detail.

**Figure 8:**
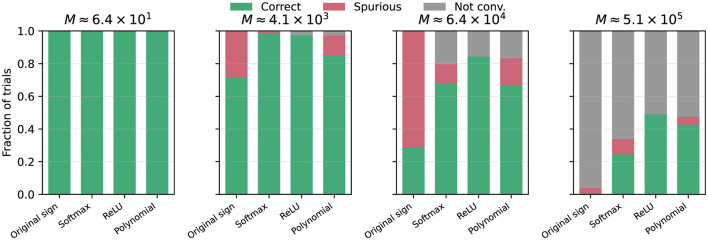
Terminal outcomes for matched-dimensionality FHRR factorization with *F* = 3 and *D*_eff_ = 1, 000.

Figure 8 decomposes the FHRR runs into terminal classes. The original complex cleanup can converge correctly at small *M*, but at larger search spaces its failures include substantial spurious convergence and non-convergence. ReLU maintains a larger correct-convergence fraction over the plotted search spaces, while softmax and polynomial cleanup show mixed behavior. These terminal outcomes clarify the accuracy curves in Figure 7: lower accuracy can arise either because the dynamics settle into incorrect complex-valued attractors or because they fail to converge within the computation budget.

### 4.6 Internal update noise

To test the stochasticity mechanism discussed by Langenegger et al., we use an internal-update-noise model. The bound query **s** is kept fixed and uncorrupted. Instead, after computing the factor-specific similarity vector:


at(j)=1DRe(X¯jrt(j)), (5)

$$\begin{array}{l} {ã}_{t}^{\left(j\right)}=a_{t}^{\left(j\right)}+\frac{\lambda }{\sqrt{D}}\epsilon_{t}^{\left(j\right)},\;\epsilon_{t}^{\left(j\right)}\sim N\left(0,I\right). \end{array}$$ (6)

The scale $1/\sqrt{D}$ matches the typical magnitude of random codeword similarities, while λ controls the internal stochasticity. This experiment tests whether stochasticity in the recurrent cleanup computation can help the dynamics escape unfavorable transient states or limit cycles, rather than whether the representation is robust to a corrupted input vector.

**Figure 9:**
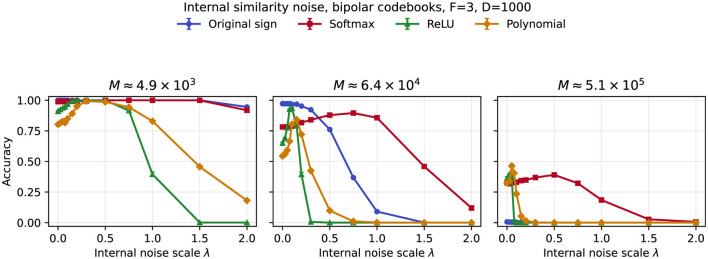
Effect of internal similarity noise for bipolar codebooks with *F* = 3 and *D* = 1, 000. The query vector is kept clean; noise is injected into the similarity vector inside each recurrent cleanup update. Error bars show standard deviations across independently resampled codebook batches.

Figure 9 reports accuracy as a function of the internal noise scale for three representative search-space sizes. At the easiest search space, sign and softmax remain close to their clean-update performance over small and moderate noise scales, while ReLU and polynomial cleanup become less reliable once the injected similarity noise is large. At the intermediate search space, moderate internal noise improves softmax relative to its zero-noise baseline, consistent with the possibility that stochasticity can help some updates avoid unfavorable deterministic trajectories. At the largest search space, softmax again shows a modest beneficial-noise regime, but all methods remain near the edge of failure and excessive noise degrades performance. ReLU and polynomial cleanup without sign projection do not show a systematic beneficial-noise effect in this experiment, indicating that the usefulness of internal stochasticity depends on both the cleanup rule and the search difficulty.

### 4.7 Convergence and empirical complexity

Each recurrent update is dominated by one similarity computation and one codebook reconstruction per factor, giving *O*(*FnD*) computational complexity. Exhaustive search scales as *M* = *n*^*F*^, so resonator methods are useful only when high accuracy is reached in far fewer than *M* recurrent steps.

Table 2 reports recurrent-update counts conditioned on correct convergence. This conditioning is essential for interpreting the table. A method can have a small number of updates among successful trials while still having poor overall accuracy if many other trials converge to spurious states or fail to converge. Polynomial cleanup illustrates this point: it can reach correct solutions in few iterations when it succeeds, but the accuracy and terminal-outcome figures show that it is less reliable across many regimes.

**Table 2:** Mean recurrent updates to correct convergence for dense bipolar *F* = 3, *D* = 1, 000 factorization.

Cleanup rule	Iters, *M*≈4.9 × 10^3^	Iters, *M*≈6.4 × 10^4^
Sign	4.2 ± 0.1	13.8 ± 1.0
Softmax	5.1 ± 0.2	19.4 ± 1.5
ReLU	6.4 ± 0.5	18.6 ± 1.1
Polynomial	3.1 ± 0.0	4.6 ± 0.7

Since all variants have the same asymptotic per-iteration complexity, empirical cost is determined by both the number of recurrent updates and the probability of correct convergence. The terminal-outcome figures, therefore, provide the necessary context for the iteration counts. In particular, fast convergence is only useful when the terminal state is correct; otherwise, low iteration count reflects rapid convergence to a wrong attractor rather than efficient factorization.

### 4.8 Effect of hyperparameters

**Figure 10:**
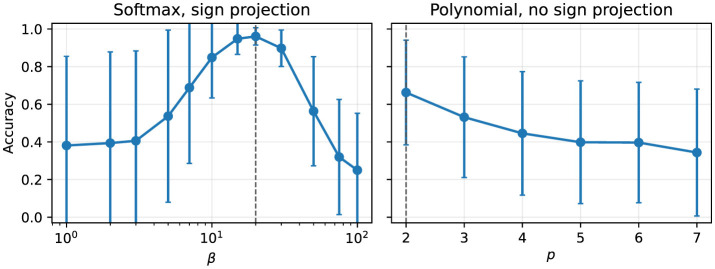
Validation sweeps for softmax inverse temperature β and polynomial degree *p*.

Figure 10 reports the validation sweeps used to select the softmax inverse temperature and polynomial degree. The softmax sweep is evaluated with sign projection, and the polynomial sweep is evaluated without sign projection, matching the selected variants used in the main experiments. Softmax exhibits a clear dependence on inverse temperature: small β values produce overly diffuse codebook weights, while very large β values can make the update too close to a hard winner-take-all rule. Intermediate temperatures give the best validation performance, as indicated by the vertical dashed line.

Polynomial cleanup behaves differently. In the no-sign-projection setting, the lowest tested degree gives the strongest validation performance, and increasing *p* generally reduces accuracy. This suggests that sharper rectification is not uniformly beneficial for resonator networks: amplifying the largest positive similarities can help only when those similarities already identify the correct factors. When the current estimates are still inaccurate, a sharper polynomial response can reinforce the wrong partial factorization.

### 4.9 Effect of sign projection

The cleanup nonlinearity and the sign-projection choice are distinct parts of the update. Sign projection maps the reconstructed factor estimate back to a bipolar state after the codebook reconstruction step. This is natural for the original sign resonator and remains beneficial for softmax cleanup across much of the plotted range, because the softmax update forms a dense weighted reconstruction that often benefits from being returned to the bipolar state space. For ReLU and polynomial cleanup, however, the cleanup weights are already sparse, nonnegative, and normalized before reconstruction. Omitting sign projection preserves graded amplitude information in the reconstructed estimate, whereas binarizing the estimate can turn weak or noisy coordinates into hard ±1 decisions.

Figure 4 isolates this effect across three factor counts by plotting the projection-off advantage directly. Positive differences indicate that omitting sign projection improves accuracy, while negative differences indicate that applying sign projection is beneficial. The effect is not uniform: for ReLU and polynomial cleanup, omitting sign projection is most helpful in the more coupled *F* = 4 and *F* = 5 regimes and for several low-to-intermediate search spaces, while *F* = 3 and parts of the *F* = 4 sweep show near-zero or negative differences. Softmax shows the opposite pattern over most moderate and large search spaces, where removing sign projection substantially lowers accuracy. We, therefore, treat sign projection as a rule-dependent design choice rather than as a fixed implementation detail. The main experiments use the selected variants: sign and softmax with sign projection, and ReLU and polynomial cleanup without sign projection.

## 5 Discussion

The experiments support viewing resonator networks through the coupled dynamics induced by their cleanup rules. The original resonator uses a sign-based cleanup rule that mirrors classical Hopfield retrieval. The softmax, ReLU, and polynomial variants are more precisely described as modern Hopfield-inspired modifications of the nonlinear cleanup stage. Softmax, ReLU, and polynomial variants modify the same basic unbind-and-cleanup loop, but the resulting coupled dynamics can have different capacity transitions and different terminal attractors. The one-factor case reduces to single-codebook associative retrieval; the multi-factor case is harder because each factor update depends on all other current estimates.

This distinction is important when importing intuition from Hopfield networks. A cleanup nonlinearity that is effective for single-memory retrieval need not dominate once the retrieval problems are coupled through VSA binding. The sign-projection ablation further shows that the projection step is rule-dependent: softmax often benefits from bipolar projection, while ReLU and polynomial cleanup can benefit from preserving superposed reconstructed states. These differences would be partly obscured if the evaluation reported only accuracy or only iteration count.

The coupled nature of resonator inference also connects this work to broader approaches for structured decomposition. For example, recent coupled-inference formulations for diffusion models treat semantic decomposition as a joint inverse problem in which factor estimates evolve together under a reconstruction constraint (Yeung et al., 2026).

## 6 Conclusion

We studied resonator-based VSA factorization through the role of the cleanup nonlinearity. By writing sign, softmax, ReLU, and polynomial variants in a common update form, we isolate the cleanup rule as the main algorithmic difference between these methods while also showing that sign projection can affect performance for some cleanup rules. The experiments show that this choice affects not only accuracy, but also the type of failure: different nonlinearities lead to different tradeoffs between correct convergence, spurious convergence, and non-convergence across factor counts, representations, hyperparameters, sign-projection choices, and internal noise levels. These results suggest that resonator networks should be analyzed as a family of coupled factorization dynamics, rather than as a single fixed algorithm, with the cleanup rule treated as key design choices.

## Data Availability

The original contributions presented in the study are included in the article/supplementary material, further inquiries can be directed to the corresponding author.
